# Simulating the Evolution of the Human Family: Cooperative Breeding Increases in Harsh Environments

**DOI:** 10.1371/journal.pone.0080753

**Published:** 2013-11-20

**Authors:** Paul E. Smaldino, Lesley Newson, Jeffrey C. Schank, Peter J. Richerson

**Affiliations:** 1 Center for Advanced Modeling in the Social, Behavioral, and Health Sciences, Johns Hopkins University, Baltimore, Maryland, United States of America; 2 Department of Environmental Science and Policy, University of California, Davis, Davis, California, United States of America; 3 Department of Psychology, University of California, Davis, Davis, California, United States of America; 4 College of Life and Environmental Sciences, University of Exeter, Exeter, United Kingdom; Durham University, United Kingdom

## Abstract

Verbal and mathematical models that consider the costs and benefits of behavioral strategies have been useful in explaining animal behavior and are often used as the basis of evolutionary explanations of human behavior. In most cases, however, these models do not account for the effects that group structure and cultural traditions within a human population have on the costs and benefits of its members' decisions. Nor do they consider the likelihood that cultural as well as genetic traits will be subject to natural selection. In this paper, we present an agent-based model that incorporates some key aspects of human social structure and life history. We investigate the evolution of a population under conditions of different environmental harshness and in which selection can occur at the level of the group as well as the level of the individual. We focus on the evolution of a socially learned characteristic related to individuals' willingness to contribute to raising the offspring of others within their family group. We find that environmental harshness increases the frequency of individuals who make such contributions. However, under the conditions we stipulate, we also find that environmental variability can allow groups to survive with lower frequencies of helpers. The model presented here is inevitably a simplified representation of a human population, but it provides a basis for future modeling work toward evolutionary explanations of human behavior that consider the influence of both genetic and cultural transmission of behavior.

## Introduction

Many animals are adapted to survive in variable and challenging environments, but only humans can make a living in such a wide range of settings, from the savannah to the tropics, from the scorching desert to the frozen tundra. Our ability to do so stems not from our physical or mental prowess as individuals, but from our ability to organize collectively, to cooperate, and to learn from one another in a manner that produces cumulative improvements and considerable diversity between human populations [1], [2]. Much theory and research into human origins is now focused on the characteristics that make it possible for human groups to generate and share such a complex culture, characteristics such as our highly developed ability to socially learn and our inclination to share resources [3], [4]. Social institutions and psychological norms of behavior have guided our ability to organize into cohesive cultural groups, shaping effective interactions both within and between groups [5], [6]. Increasingly, human parenting behavior, which has been described as “cooperative breeding” because allomaternal parental care is essential for the raising of human young, is recognized as having played an important role in the evolution of these characteristics [7]–[11]. Humans have a number of adaptations that facilitate the creation and maintenance of family groups whose members contribute to the raising of young. For example, humans have a unique life history; it is common for females to live many years after they have ceased to be fertile [12]. Thus human families usually include at least one experienced mother with time to help look after other women's children. The longevity that makes grandparenting possible is almost certainly underpinned by genetic adaptations. However, other adaptations that serve to ensure mothers have help raising young appear to be mostly cultural, such as the marriage customs through which a sexual relationship and responsibility for offspring are formally acknowledged. Caspari and Lee [13] have also argued that the increased longevity of modern humans was driven primarily by cultural and demographic factors rather than genetic factors, as indicated by the human fossil record.

Understanding the features and dynamics of family structure and group organization, with attention paid to lifespan stages, is essential for gathering a complete picture of how modern humans evolved. It is increasingly recognized that theoretical models complement empirical work on evolution. Models provide a formalization of theory and boundary conditions for hypothesis formation. Analytical models typically focus on one small piece of the ecological puzzle, while ignoring many environmental, structural, and psychological details. This is partly for the purposes of tractability but also in the interest of simplicity, both of analysis and of parsimony. Nevertheless, the use of more complex computational models can shed light on processes of organization and evolution that are missed by simpler models. Spatial structure, temporal changes in social networks, lifespan-dependent aspects of individual behavior, or extensive heterogeneity in the population play important roles in the historical and evolutionary trajectories of human groups.

A striking example of the importance of including heterogeneity comes from a computational modeling study of the Anasazi, a now extinct society native to the North American Southwest [14]. A detailed agent-based model was built that was able to re-create details of the population expansion and subsequent collapse. However, the re-creation was only possible when age onsets for fertility and death were made heterogeneous across agents. Thus, a complex computational model may be justified when details such as individual heterogeneity and sociospatial structure are important to the dynamics under investigation.

Recently, Smaldino, Schank, and McElreath [15] presented a spatial agent-based model of the evolution of cooperation in harsh environments, in which increased environmental harshness was modeled as an increased energy deduction (a “cost of living”) incurred by each agent at each time step. The model demonstrated that, although harsher environments caused cooperators to fare worse than freeloaders in the short run, individuals with the highest fitness were observed in emergent groups with high concentrations of cooperators. Harsh conditions led to local extinctions in regions where cooperators were scarce or not well assorted. Most importantly, harsher environments led to higher long-term frequencies of cooperators, lending theoretical support to Kropotkin's [16] proposal that harsh environments should select for cooperation. Although harsh environments have previously been shown to select for reduced parasitism [17], [18], this was the first model to show an increase in cooperation with increased environmental harshness in which cooperative acts yielded positive payoffs and defection consistently outperformed cooperation in single interactions. These results depended on a model that incorporated aspects of life history (mobility, varied social partners, decoupled birth and death) and spatial structure, and as such contained somewhat more biological realism than many game theoretic models. Nevertheless, the model was still abstract in many ways; sociospatial structure arose through random movement and resources were obtained only through pairwise interactions.

The study of recent human evolution, including our cultural evolution, is likely to benefit from the development of a family of models which are even more complex, incorporating aspects of individual life history, social structure, social learning, and behavioral institutions. Ideally, these models would be unified by a common framework, which could be modified or adapted to investigate a wide range of questions about how the evolution of humans and their groups (families and wider groupings) resulted in an animal with the characteristics we observe in modern humans. The model we present in this paper is our first step toward developing such a framework. It incorporates representations of many elements of human life history and social structure found in small-scale societies [19]. We use this model to study the evolution of cooperation in a context which has a direct effect on fitness: in the caring of young. We then define environmental harshness as increasing costs of raising young relative to the availability of resources.

Below, we will first discuss the role of cooperative breeding in human evolution in more detail, followed by an overview of our model structure. We will then present the model in more detail. We will show that this more realistic model replicates the findings of the previous model, but also illustrates how factors such as genetic adaptability and seasonal variability in available resources complicate the picture. We will conclude with a broad discussion of future directions.

### Human Cooperative Breeding

We can define *cooperative breeding* behavior as individuals exerting costly parenting effort to contribute to the developmental success of an infant or juvenile that is not their own offspring [20], [21]. Among terrestrial vertebrates, this behavior is more common in birds than mammals but has evolved independently in a number of mammalian families [22], [23]. Several authors argue that humans can be considered cooperative breeders because, although parenting behavior is highly culturally variable, in no culture do mothers raise their children without help from others [7]–[10], [24]. Hill and Hurtado [24], in their studies of contemporary South American hunter-gatherer societies, found not only that cooperative breeding behavior was ubiquitous, but also, crucially, that husband-wife pairs were physically incapable of procuring sufficient food for their offspring and themselves without help from others. Moreover, they found that meat acquisition of Ache hunters over a given 90-day period was often highly variable for any given individual, as a result of illness, injury, or luck. Sharing food resources between nuclear families was therefore necessary to ensure the survival of young children.

These observations suggest that humans are more accurately described as “cooperative breeders” than “biparental carers” as has been suggested by several influential evolutionary psychologists (e.g., [25]). Research in a number of small-scale subsistence communities has shown that the death or absence of a child's father often had no effect on the survival or welfare of the child [10], [26]. Presumably, in these subsistence societies contributions from the child's other kin and individuals who are unrelated but allied with the family are able to compensate for the lack of paternal contributions. For many parents living in economically developed large-scale societies, extended kin groups are no longer essential for raising children, but technology and the extensive networks of cooperation and division of labor in these societies have reduced the physical effort required for acquiring food. Also, state institutions such as education and health care systems provide considerable assistance to mothers raising their children.

It is likely that hominins have been raising their children cooperatively for some time [8]. The large brains of humans inevitably make our offspring costly to raise because the growth and development of brain tissue requires high levels of energy and nutrients [27]. Van Schaik and colleagues have argued that the increased encephalization of the hominin line would not have been possible unless females were receiving help provisioning their young [28]. In particular, cooperative breeding has been identified as a potentially crucial factor in the evolution of human prosociality and our tremendous cognitive advantage over our nearest relatives, the great apes [8], [29]. Early Homo fossils are also the earliest hominin fossils to be found associated with the drier, more heterogeneous environments which began to expand in Africa at the beginning of the Pleistocene [30], [31]. In these harsher environments natural selection might have favored cooperation in the raising of young. Scarcity of water sources would have placed considerable stress on lactating females because human milk, like the milk of all primates, is very dilute [32], [33].

### Environmental Harshness and Cooperative Breeding

We present a model that includes certain key aspects of human life history and social structure for modern humans that are likely to have existed during the time of the last glaciation. This was a time of climate instability that paleoclimate data suggests was more extreme than that which occurred earlier in the Pleistocene [34]. It was also during this time that *Homo sapiens* began to spread out of Africa, and the first evidence of the sustained accumulation of highly complex culture appears. To have survived in these conditions, human groups would have had to be nomadic hunter-gatherers, and we envisage that they would have had the egalitarian norms observed in contemporary nomadic hunter-gatherers [35]. Research on such societies suggests that this egalitarianism reduces mating-related competition among males to a modest fraction of their total acquired resources [36].

Humans have a number of physical adaptations to harsh tropical and subtropical environments such as savannas and deserts. For example, our lack of body hair, increased ability to perspire, and the dark skin pigmentation still found among humans living in such climates likely facilitated the exodus from the forest to the savanna [37]. The subsequent migration to cooler temperate and subarctic environments presented a suite of new adaptive challenges – adequate clothing and shelter, storage of food against seasonal shortfalls, and the need to rely more on hunting due to fewer edible plant resources. Expansion into such environments also likely necessitated an increased reliance on sharing time, attention, and food resources in the rearing of children, including genetically unrelated children [24], [29]. To judge from the size of language groups, extra-tropical people often had larger societies, perhaps to cope with greater environmental harshness and increased risks [38]. Male contribution to the diet increases with increasing latitude [39]. Larger group sizes may also have been necessary to sustain more advanced technologies required in harsher climates [40] and avoid catastrophic losses in technologies seen when populations suddenly shrank [41]–[44]. Thus, the evidence suggests that many adaptations to harsher environments as humans left Africa were cultural in nature, concerning both individual behavior and group organization.

Our model will focus on how cultural norms of cooperative breeding spread in a population of agents through the process of natural selection when direct transmission is cultural rather than genetic (i.e., cooperative behavior is learned). We assume that as environmental harshness increases, so does the effort that must be exerted to successfully raise an infant to adulthood (i.e., effort exerted in providing food, care and attention). We will then explore how additional factors such as constraints on the ability to genetically adapt and yearly variability in environmental harshness mediate the evolution of cooperative breeding.

There are fitness costs associated with contributing alloparenting effort, which should be greatest for reproductive age females. Those mothers who contribute effort to raising other females' children will be able to devote less effort to raising her own. In the case of males and post-reproductive females, the costs will be smaller, if not nonexistent. Devoting more effort to parenting will increase risk of injury and disease, and thus reduce life expectancy. Cooperation is also likely to reap benefits, however, such as when a hunter is given preferential access to court a young woman after sharing food with her mother and sisters. For males, contributions to cooperative breeding will tend to come from reduced contributions to mating effort. Many processes may explain how evolution solved the dilemma of cooperation inherent in reduced male mating competition in our species [45]. Our future work will include a comparison of culturally transmitted strategies to adjust the costs and benefits of contributing parenting effort. In this introductory model, we will assume that cooperation is costly for reproductive-age females but that there is no net cost for other cooperating adults.

## A Model for the Evolution of Human Family Groups

This section provides a basic description of the model. More technical details following the ODD protocol for describing agent-based models [46] can be found in Appendix S1. The design of our model is as follows. Individuals live together in family groups, which compete for resources as the overall environment has a finite carrying capacity. Within each group are children and adults delineated by sex, age, genetic quality (a measure of their ability to acquire resources and stave off infection), and whether or not they are a cooperator. Children are linked to their mothers, who must acquire sufficient resources through their own efforts and the contributions of the members of their group to raise their children to adulthood. Upon leaving childhood behind, young adults can begin to acquire resources of their own, and thus may contribute to the raising of children within their family group. Young adults also attempt to find a mate. When a marriage occurs, the female leaves her natal family group and joins the group of her husband. Each year, cooperators contribute substantial effort to collective childrearing. Uncooperative “freeloaders” contribute much less. If a mother cannot acquire enough resources to care for one of her children, the child dies. Death can also occur from illness; the risk of succumbing to illness is linked to a genetic trait, which a child inherits from her parents. An individual's genes reflect her physiological adaptedness to her environment, and so also affect the amount of effort (in terms of food, time, and attention) she can provide toward childrearing. Whether or not an individual becomes a cooperator is learned during childhood from the adults in the natal family group.

Individuals in our model have a life course consistent with the life history observed in contemporary foraging populations [19]. They are born, grow up, mate, produce young, and die within family groups. Individuals are assigned the following characteristics:

1. Sex, assigned at birth with 50 percent probability of being female.
2. Age, 0 to 100, with chance of mortality changing over the lifespan. Children become adults at the age of 18, and adults become non-reproductive elders at the age of 50.
3. Family group membership, assigned at birth but, for females, may change at marriage as females join the family groups of their mates.
4. Genetic quality, a continuous variable that influences the chances of survival and ability to gain resources, assigned at birth and based on mean of genetic quality of an individual's parents plus error.
5. Cooperativity, a socially learned characteristic assigned to agents when they reach adulthood. The probability of being a cooperator is equal to the frequency of cooperators in one's natal family group at age 18. Adult cooperators contribute a large portion of their resources to the family group for the support of offspring.

Family groups consist of children, unmarried adults, parents, and elders. All individuals become one year older at each time step and follow a lifecycle that reflects the acquisition and consumption of resources and mortality risks observed in hunter-gatherer groups [47]. Child-rearing imposes a cost on family groups because children require care and provisioning. This cost is paid by the child's mother, and by alloparental contributions from the group. In harsher environments, alloparental contributions are required to sustain childrearing. The cost is maximal at birth and gradually decreases as a child needs less care and learns to acquire resources for itself. At 18, children become adults and cease to be a cost to their group, beginning instead to contribute resources to support children in the family group. Individuals' contributions increase as they age until mid-life (the age of elderhood) and then decline. Each year, agents have a risk of dying. The risk is high in the first year and then decreases, remaining stable throughout adulthood and then increasing again past the age of elderhood.

Each individual has a *genetic quality, q*, which reflects the degree to which his or her physiological characteristics are adapted to the environment. This adaptiveness includes the ability to ward off illness and to secure available resources that could be contributed for childrearing (e.g., food, time, and attention). Genetic quality is represented by a real number between 0 and 1 and is an inherited trait derived from the genetic qualities of an individual's parents (see details in Appendix S1). To simulate a population's migration into a novel and harsh environment, we can limit individuals' ability to evolve high levels of genetic quality, resulting in the need to adapt culturally. This reflects the observation that cultural traits can evolve much more quickly than genetic traits [48], [49], which accounts in part for the tremendous success of the human species across a wide variety of environments.

Individuals also have a binary *cooperativity* trait (i.e., they are either a cooperator or a defector), which represents their willingness to contribute resources to their family group toward the raising of children. An individual is assigned this trait at the age of adulthood with a probability equal to the proportion of cooperator adults in his natal family group. So the cultural transmission of cooperative behavior is *unbiased* in the sense that the probabilistic method we describe is mathematically equivalent to choosing an individual at random and copying her, but *biased* in the sense that that random individual is selected only from within an agent's natal family group [48]. The assumption that cooperative behavior is socially learned and unbiased within an agent's family group has an important consequence: cooperation will increase if families with more cooperators have more offspring, regardless of whether the parents of those offspring are cooperators themselves. We made this assumption for simplicity but it can be relaxed in future models aimed at assessing the influence of more complex mechanisms of cultural transmission.

In addition to cooperating, kin and other social relations may also compete with one another for resources [50], [51]. To some extent, this is endogenously captured in the model by the fact that family groups have limited carrying capacities. It is well known that kin effects may also aid the evolution of cooperation. We do not model kin effects explicitly, but because family members will tend to be more closely related than individuals of different family groups, kin-biased cooperation is endogenously driven through the mechanism of positive assortment [52].

**Figure 1:**
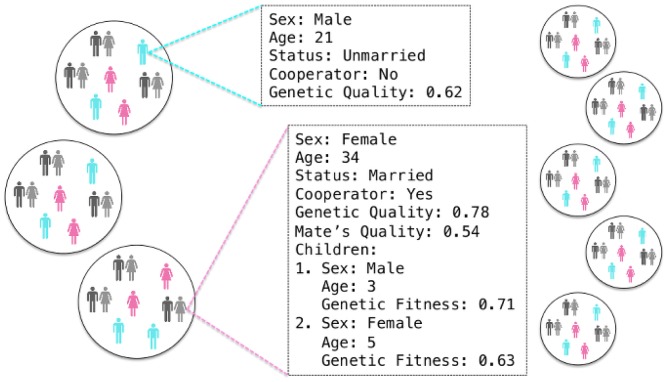
An illustration of the individual and group structure of the model. There are a number of family groups, each of which contains a unique set of agents. Unmarried agents are colored, married agents are grey. Because children's lives are attached to their mothers' until they reach adulthood, children are not shown. Each agent is characterized by sex, age, marital status, cooperativity, and genetic quality.

Each simulation was initialized so that each family group had an equal number of individuals of each sex and equal numbers from each of the four age categories (children, unwed adults, parents, and elders) whose ages were randomly assigned within the associated limits. Individuals with higher or lower genetic quality and cooperativity were randomly distributed in the population so that some families started with higher mean genetic quality and a higher proportion of cooperators than others. An illustration of the population structure is given in Fig. 1.

Once initialized, the model schedule proceeds by performing the following five stages in order at each time step (notionally a year):

1. *Matchmaking*: Unmarried adults search for mates and, if paired, females migrate to their mate's family group.
2. *Family Fissioning*: Large family groups split and spread into unused territory, if available. If no territory is available, groups don't split.
3. *Resource Contributions*: Adults contribute resources toward helping eligible females raise children, with cooperators contributing considerably more.
4. *Childbirth, Childrearing, and Child Death*: Married females with sufficient resources produce offspring, and children die due to lack of resources, illness, or chance events.
5. *Adult Aging and Death*: Adults age and eventually die.

### 

#### 1. Matchmaking

Unmarried adults are randomly paired with an unmarried member of the opposite sex from another family. An individual *i* agrees to marry individual *j* with a probability that increases with *j*'s genetic quality, reflecting the increased desirability (attractiveness) of mates with “good genes” [25], [53], [54]. If both agree to the match, the female moves to the family group of her spouse. If either does not agree, the individuals remain unmarried. As individuals age, the strictness with which preferences constrain the acceptance of a potential mate decreases, so older agents are more willing to marry agents of lower genetic quality. This mate choice rule generates assortment for genetic quality [55] while ensuring that most agents eventually find a mate. Full details of the mating algorithm can be found in Appendix S1. We assume that individuals use relatively simple heuristics, rather than complex optimizing strategies, in selecting a mate. This is based on research that suggests not only that humans often employ simple decision strategies, but also that these heuristics are often quite effective across a wide range of conditions [56], [57]. We note that other criteria not included here may be involved in mate choice procedures, including homophily, family reputation, and individual personality traits such as charm and agreeableness. Our modeling framework has been developed so that different mating strategies can be easily implemented in future investigations.

#### 2. Family Fissioning

Family groups live on patches, each of which has a maximum carrying capacity. When a family size exceeds half the patch carrying capacity, the group attempts to split if there is a free patch available, a situation that occurs when a patch's previous occupying group has died out due to an inability to raise sufficient children to maturity to offset their local death rate. If fissioning occurs, males and females from each class of adults (unmarried adults, parents, and elders) divide evenly between the two new family groups. Children accompany their mothers. Individuals are therefore subject to group-level selection. Successful family groups flourish as unsuccessful groups become extinct.

#### 3. Resource Collection and Contribution

To raise children, a mother needs more resources than she can collect on her own, so the raising of children requires resource contributions by other family members [24], [47]. The amount of resources available to an individual at a given time is determined by her age and genetic quality. Individuals contribute some of their collected resources to the married reproductive-aged females in the family group for re-allocation to children. The proportion of resources contributed is determined by whether or not one is a cooperator. Cooperators contribute 90 percent of their resources, defectors contribute only 10 percent. Keep in mind that because resources are divided among only the child-bearing females, such females who are also cooperators often recoup resources in excess of their contributions as long as there are sufficient cooperators in their family group. Still, within groups non-cooperative mothers will have relatively more resources to contribute to their offspring than will cooperative mothers. We assume that the resources available for donation are in excess of what an individual needs for personal survival, and for simplicity, non-donated resources kept by males and women without dependent children does not influence their chance of survival. Future work will explore the trade-offs of differential resource usage for other fitness-affecting purposes.

#### 4. Childbirth, Childrearing, and Child Death

Reproductive-age females allocate the resources contributed to them to the production and rearing of offspring. Those who already have children first allocate resources to sustain them. If there are insufficient resources, the youngest child dies, with the next-youngest dying if there are still insufficient resources for the remaining children, and so on. The base cost of childrearing, *β*, is equal to the cost of giving birth. The cost of supporting a child in any one year is derived from the baseline cost of childrearing, *β*, and decreases as the child ages (see Appendix S1 for details). The value of *β* is a metric of environmental harshness, representing the costs of childrearing relative to the resources available to the population. If a married adult female has more resources than needed to sustain her children (if any), she can reproduce if the size of the family group she belongs to is less than the patch's carrying capacity. The genetic quality of a newborn child is the mean of its parents' genetic quality plus error. Newborns are equally likely to be male or female. Each child has a nonzero probability of dying each year, determined by its age and genetic quality. Children who survive 18 years become adults.

#### 5. Adult Aging and Death

Each adult agent also has a nonzero probability of dying each year, which is a function of its age and genetic quality. If an agent does not die, it continues to age. Agents who survive 50 years enter *elderhood*, after which the females no longer produce offspring (and neither do the males, as, for simplicity, we assume all mating occurs within marriage), and the probability of death for both male and females begins to increase. If an agent survives 100 years, its probability of death is one.

For the simulation runs presented below, the initial genetic quality of the population was normally distributed with a mean of 0.5 and a standard deviation of approximately 0.24, capped between 0 and 1. The initial frequency of cooperators in the population was 0.5 unless otherwise stated. Our results are averaged from 50 runs of each condition, each of which was run for 10^4^ time steps.

## Results

### Harshness due to increased costs of childrearing

**Figure 2:**
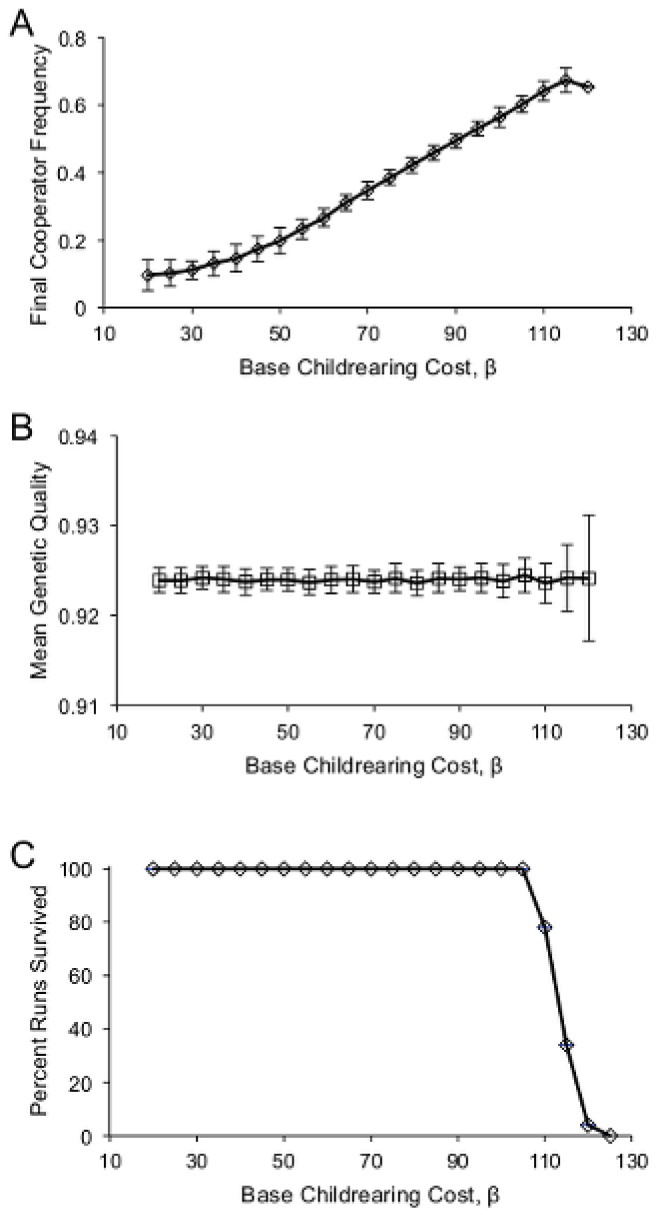
Long-term results of the model at *t* = 10^4^, as a function of the baseline cost of raising a child, *β*. (A) The population cooperator frequency, and (B) the mean genetic quality in the population. Error bars are standard deviations. (C) The percent of runs in which the population did not go extinct.

**Figure 3:**
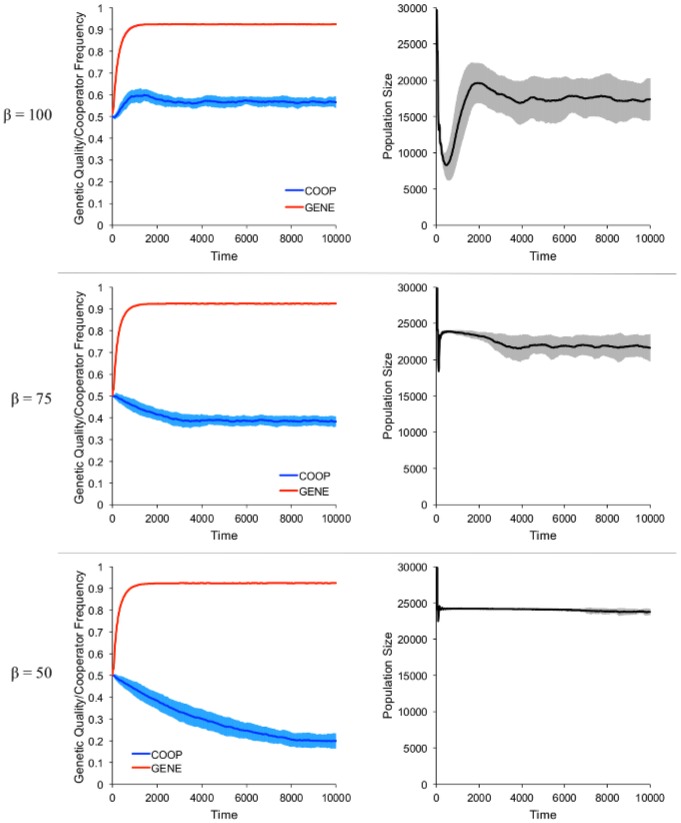
Model dynamics. The three rows reflect three different costs of childbirth. The left graphs reflect the average genetic quality in the population (in red) and the frequency of cooperators (in blue). The right graphs are the total population size. The dark lines are averages across 50 runs (or all runs in which the population did not go extinct), the shaded regions are standard deviations.

Environmental harshness, in the form of increased costs of childrearing, was positively correlated with the long-term frequency of cooperators (Fig. 2A) but not with the genetic quality of the population (Fig. 2B). Genetic quality was associated with survival as well as resource production, and so was always positively selected for irrespective of the costs of childrearing. Figure 3 shows the model dynamics for three levels of environmental harshness. Genetic quality increased in all cases, but the frequency of cooperators increased only in the top row, when the cost of childrearing was greatest (*β* = 100), and fell in the other two cases, with a larger fall associated with lower costs. High childrearing costs also led to an early dip in the population size, when the family groups with few cooperators died out. This was followed by a recovery as the surviving family groups expanded. When the cost of childrearing was too high, recovery from this population dip was sometimes impossible, leading to complete population collapse (Fig. 2C). Note that while the genetic quality in the population rose rapidly due to relatively high rates of mutation, the overall results were qualitatively unchanged when the mutation rate was lowered and hence genetic quality took longer to increase.

These results extend the findings of Smaldino et al. [15] and show that the theory of the evolution of cooperation in harsh environments developed therein – regions with few cooperators perish, leading to the survival of groups with higher number of cooperators as environmental costs increase – is robust in the sense described by Levins [58]. We show here that this result holds regardless of whether cooperation is transmitted vertically from parent to offspring or socially learned though unbiased transmission from an individual's larger social group.

Genetic quality increased to approach its maximum value regardless of environmental harshness (transmission error kept it from reaching unity), but even when the cost of childrearing was at its highest, the long-range frequency of cooperators was never greater than around 0.7. Cooperator frequency rose to its highest peak early in each run (if it rose at all) and fell back as the mean genetic quality of the population increased because individuals of higher genetic quality were able to acquire more resources.

**Figure 4:**
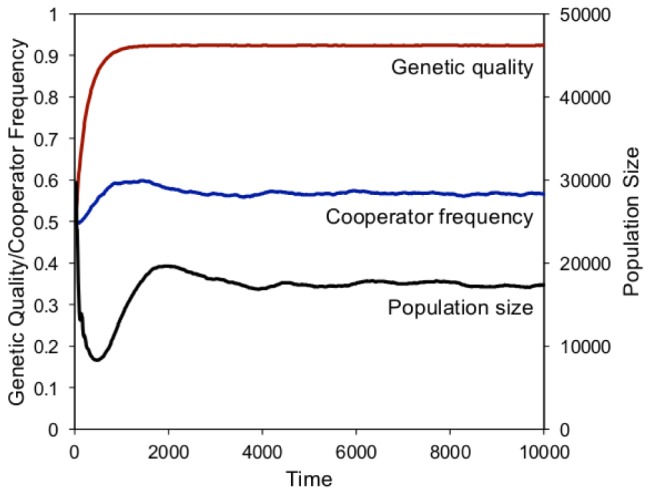
Model dynamics under high costs of childrearing(*β* = 100).

When the cost of childrearing was high, the population fell sharply during the first few generations as family groups with fewer cooperators and/or lower genetic quality perished. Only groups with a minimum threshold of cooperators could survive, so the frequency of cooperators increased rapidly. Eventually, though, as natural selection increased the genetic quality in the population, family groups needed fewer cooperators, and individual-level selection against cooperators decreased the cooperator frequency. The top row of Fig. 3 is consolidated and summarized in Fig. 4, which highlights the dynamics under high costs of childbirth.

### Harshness due to reduced genetic adaptability

In the baseline model, we assumed that, through the process of natural selection on genes, individual physiologies could adapt quite well to their environments, and maximize an individual's ability to acquire resources and stave off infection. The generality of this assumption is limited, however. As anatomically modern humans spread out of the tropics into colder, harsher environments, it is unlikely that they could quickly adapt genetically. Indeed, even today, humans who live near the Arctic Circle require a host of cumulative cultural innovations and ingroup cooperation in order to survive in the tundra [1]. Here we considered limitations to individuals' ability to evolve their genetic quality. To do this, we imposed a maximum genetic quality *γ*≤1, and initialized the population with a mean genetic quality of *γ*/2. The initial model presented above was recovered when *γ* = 1.

**Figure 5:**
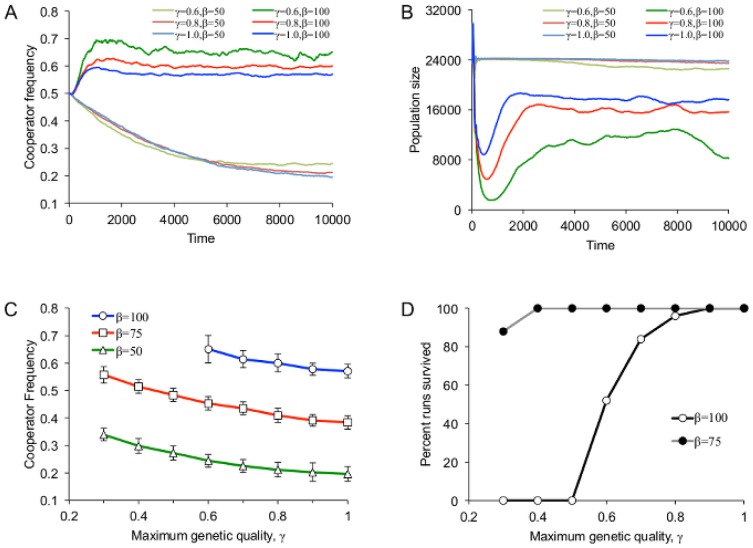
Model dynamics and long-term outcomes for varying maximum genetic quality, *γ*. (A) The mean cooperator frequency and (B) population size as a function of time for several values of *γ* and *β*. (C) The mean ±SD cooperator frequency as a function of *γ* for several values of *β*. (D) Percent runs survived as a function of *γ* when *β* = 100 and *β* = 75.

In all cases, the previously described patterns of an early increase in cooperation followed by a small decrease and stabilization were seen whenever the cost of childrearing was high, and a decrease in cooperation followed by a stabilization was observed when the cost of childrearing was low (Fig. 5A). These results were also accompanied by an early drop in the population size followed by recovery and stabilization in the former case, and an absence of a drop in the latter case (Fig. 5B). Predictably, the organism-environment adaptive fit also affected the evolution of cooperation. In particular, the long-term frequency of cooperators was higher when *γ* was lower, since more cooperation was needed to survive. When the cost of childrearing was high, lower *γ* led to more dramatic initial dips in the population size and to smaller long-term population sizes at recovery. Figure 5C summarizes the relationships between maximum genetic quality, cost of childrearing, and cooperator frequency. The former two appear to have additive influence on the latter, which makes sense because both factors directly influence the relative contributions needed for childrearing. Figure 5D shows the proportion of runs for which the population completely collapsed as a function of *γ* for the cost of childrearing *β* = 100 and *β* = 75. All runs survived for *β* = 50.

### Harshness due to variability in the costs of childrearing

An environment may be harsh in the sense that a key resource, such as water, is in short supply. As long as the environment is stable, however, organisms can develop adaptations to cope with environmental limits and this will have the effect of reducing harshness. Highly variable environments remain harsh because static adaptations, whether genetic or cultural, cannot keep up with environmental changes. For example, the availability of resources may vary if the climate is highly volatile or if competition between species causes rapid fluctuation in population sizes. As a result, both the absolute and relative fitness of individuals will fluctuate in turn. Environmental variability can be an important force in population collapse, as the struggle for existence increases and individual adaptations are no longer adequate or well-honed for survival [40], [59], [60].

The presence of cooperative breeding in some non-human species has been observed to correlate with temporal variability in environmental conditions [61], [62], which supplements the intuition that environmental variability may have contributed to cooperative breeding practices in humans. To test this in the context of our model, we added a noise term *ν* to the cost of childrearing (which forms the basis for the cost of rearing children of any age), so that in any given year, the new cost was
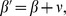



where *ν* was a random number drawn from a normal distribution with a mean of zero and a standard deviation *σ_ν_*. This variability in the cost of childrearing represents yearly environmental fluctuations in the proportion of resources gathered that are required to successfully rear offspring. It is worth noting that there are many types of environmental variability, and many ways to model them [48], [60], [63], [64]. Our intent here was not to include a comprehensive analysis on cooperation in variable environments, but rather to show that such variability could be easily added to our model and to assess the influence of one such instantiation on the evolution of cooperation.

**Figure 6:**
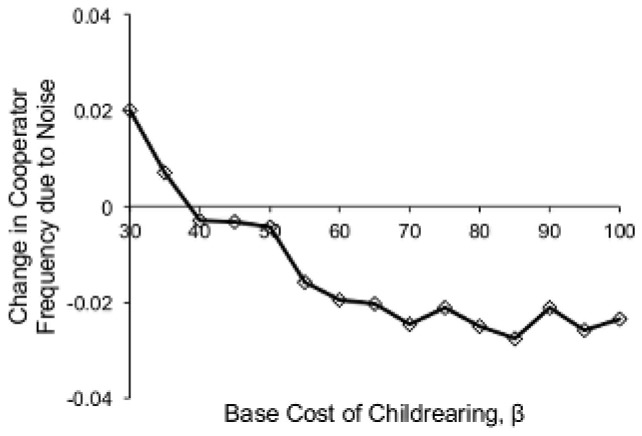
Variability in the costs of childrearing. The difference in cooperator frequency at *t* = 10^4^ between noisy and noiseless environments, as calculated by the mean cooperator frequency without noise subtracted from the mean cooperator frequency with noise. For these runs, *γ* = 0.8 and *σ_ν_* = 10.

We found that, counter to our expectations, yearly variability in the costs of childrearing led to a small *decrease* in the average long-term frequency of cooperators in the population as long as the average cost of childrearing was moderate-to-high (Fig. 6). This is because children are most costly at birth, and the relative cost of childrearing decreases as children age. Groups that lack sufficient resources to produce new offspring during an average year may nevertheless do so during a year when the costs are below average. Although costs will also rise above average with equal likelihood, children born during a low-cost year will by this time be older and therefore less costly. In this way, groups can survive with fewer cooperators when costs of childrearing are variable than when costs are constant. This decrease in cooperator frequency due to variability is, however, quite small compared with the increases due to higher costs of childrearing and diminished adaptive fit, as seen by comparing Fig. 6 with Figs 2A and 5C.

For very low average costs of childrearing (*β*<40), variability led to a small increase in cooperator frequency. However, this result is misleading. Very low values of *β* produce a floor effect, because *β*' cannot drop below zero. This means that when the average cost of childrearing is very small, the expected value of *β*' will be greater than *β*, and it is this increase in the average cost of childrearing, rather than variability per se, that drives the increase in cooperator frequency.

### Initial conditions and population structure

**Figure 7:**
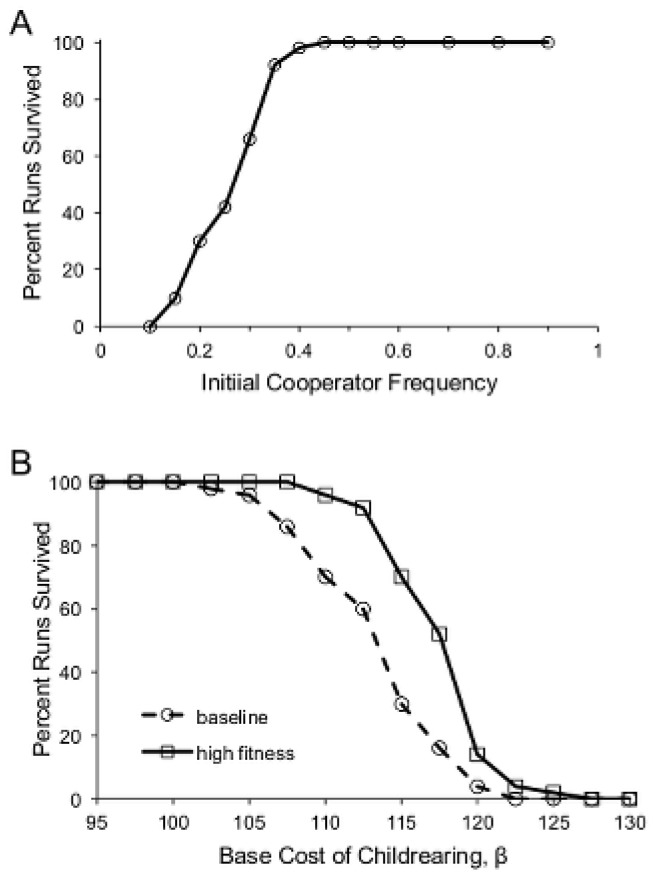
Influences on population survival. (A) The percent of runs in which the population survived as a function of the initial cooperator frequency, for *β* = 100. (B) The effect of both high cooperator frequency and high genetic quality on population survival for high costs of childrearing compared with the baseline model. Initial conditions in the “high fitness” condition were a cooperator frequency of 90% and a mean genetic quality of 0.86, compared with 50% cooperators and mean genetic quality of 0.5 in the baseline condition.

All of the results presented so far are based on runs initialized with 50% cooperators. However, if we assume that the start of a simulation run represents a sudden increase in environmental harshness (such as that which must have often been experienced by our ancestors during the climatic instability of the most recent glaciation), it may be more realistic to assume that cooperators are initially rare. We therefore tested the model's robustness to different initial cooperator frequencies. We found that the long-term results were largely insensitive to the initial cooperator frequency. Neither the frequency of cooperators nor the mean genetic quality at *t* = 10^4^ were influenced at all by the initial cooperator frequency. In other words, if cooperators were initially rare, their numbers increased, and if cooperators were initially common, their numbers decreased. The initial cooperator frequency was, however, an important factor in whether the population survived the early crash when the cost of childrearing was high (Fig. 7A). With too few cooperators, family groups could not muster the resources to survive. This suggests that even with relatively low average costs, high levels of cooperation would be favored by group selection if environments occasionally experienced severe increases in harshness.

If, on the other hand, the population was generally more cooperative and consisted of individuals of higher genetic quality, it should be better able to withstand population “crashes” (see Fig. 3) and hence survive in harsher environments. To test this, we initialized the population with 90% cooperators and with a modal genetic quality equal to 1.0 (with a mean of 0.86, achieved by setting <*g_i_> = ĝ*, see Appendix S1). As expected, the population was able to weather harsher storms (Fig. 7B), even though the long-term frequency of cooperators was unaffected (among runs in which the population survived).

Our results were also robust to changes in the population structure, including fewer family groups and a smaller maximum family size (in either case the total population size was smaller, and therefore more fragile). For the former, we tested 30 vs. 80 family groups. For the latter, we tested a maximum family size of 150 vs. 300. None of these factors had any qualitative effects on our results. Our results were also unchanged if males rather than females left their natal groups at marriage.

## Discussion

We have presented a model of social evolution in a population of agents with a family group structure and individual life history roughly similar to that of humans living in small family-based communities – the kind of social environment in which almost all humans lived for most of their evolutionary history. The model examines the effect of environmental harshness on the frequency of a socially learned trait, to be an alloparent. The findings build on those of Smaldino et al. [15] and show that harsh environments select for a long-term increase in cooperation but in conditions based on assumptions that are more realistic and related to human biology and social structure. In this case, cooperation was specific to the context of raising children, and environmental harshness was defined as either an increase in the costs of childrearing or as a decrease in the ability to genetically evolve adaptive traits. This second definition of harshness is particularly pertinent to the evolution of social norms and institutions as anatomically modern humans spread throughout the globe starting in the Paleolithic, because the evolution of genetic traits is a relatively slow process compared with the potential speed of cultural evolution [48], [49]. The ability to learn new behaviors and transmit them through social learning, particularly when those behaviors are related to widespread cooperation, has likely been one of the most significant factors in the success of the human species [1], [3].

Variability in resource availability (or, equivalently, variability in costs requiring the use of those resources) may be characterized as another form of environmental harshness. Certainly, it increases the uncertainty faced by an organism, and can introduce complex selection dynamics, since what is most adaptive may change from year to year [65]. Our results show that normal variation in the yearly cost of childrearing can, counterintuitively, decrease the need for cooperation. This effect was driven by the fact that the costs of rearing a child decreased as the child aged. As far as we can tell, this is a previously undocumented “economic” phenomenon: when the investment cost for a resource decreases over time, variation in the available investment capital facilitates the development of that resource. Nevertheless, cooperation in many species appears to increase under increased resource variability [61], [62], which should give us pause in the interpretation of these results. Yearly variability in resources may be better modeled as a skewed distribution in which the costs of childrearing do not substantially decrease from the median values, but in which occasional catastrophic events can significantly raise those costs. In these cases, the mean cost of childrearing would increase relative to the median cost, which our model predicts would increase cooperation. Furthermore, our model does not account for variability on shorter time scales, such as when some individuals fail to acquire resources due to illness, injury, or luck.

### Limitations and Future directions

Like all such models, this one provides only a rough approximation of conditions existing in a small-scale human society. The challenge faced by all modeling endeavors is that, to achieve meaningful results, simplicity and parsimony must be balanced by sufficient complexity and realism [66]. This model allows us to observe effects in a system in which natural selection is working on both individuals and groups (families) when characteristics can be transmitted both genetically and through social learning. Because of this, its basic framework has the potential to provide a better way of testing ideas about human evolution. It allows us to assess the long-term effects on fitness of variations in individual-level traits (transmitted genetically or through social learning) and group-level traits, such as social institutions.

We envisage this model to be the basis of a family of models into which parameters can be introduced and their values adjusted depending on the aspects of human behavior, biology or social organization which are of interest. Most mathematical and verbal models of the evolution of social traits consider costs and benefits only in terms of competition between individuals for food, for mates, or for the survival of offspring (e.g., [67], [68]). While this is valid in non-human species, it does not take into account the fact that human groups use socially transmitted institutions of reward and punishment to manipulate the costs and benefits its members face. These institutions are group-level characteristics and they are subject to natural selection because groups with the more effective institutions will be more successful [2], [6]. Thus, an explanation of the evolution of our unique species requires more complex models, accounting for details at multiple levels of organization and for the coevolution of genes and culture. We have presented this model as the first stage in an effort to create models that capture aspects of the processes of human social evolution that are underrepresented in the current literature, with a particular focus on the importance of cooperation in the context of childrearing. Painting a fuller and richer picture with future modeling work will likely require the incorporation of additional details and nuances of human life history, social institutions, and social structure.

One noteworthy limitation was that our model did not investigate sex differences related to social learning and cooperativity. In our model, males and non-reproductive females did not incur any costs for cooperating, leading to neutral selection on cooperativity at the individual level. This contrasts with the negative individual-level selection on cooperativity for reproductive females (cooperation was always favored at the group level). Cooperative strategies, however, were socially learned via unbiased transmission by averaging across the strategies of all adults in the family group, regardless of sex. This obscures differences in fitness for male and female cooperators, and also ignores evidence suggesting that, in at least some contexts, children preferentially learn from adults and other children of their own sex [69]–[71]. An important avenue for future research is the investigation of sex-biased learning strategies and the emergent differences in male and female patterns of cooperativity.

Even more important is deeper investigation into the cooperative dilemmas associated with cooperative breeding and how they are managed in humans. The term “cooperative breeding” is used to describe the parenting behaviors of many animal species, but discussion of how these relate to theoretical concepts of cooperation is only beginning [72]. In humans, no forms of cooperation, including cooperation in the raising of young, can be understood without considering culturally evolved traits and their effects at the individual and group levels. Models such as the one we describe here, which look at the coevolution of genetic and cultural traits, will play an important role in developing this understanding. Our modeling framework has vast potential for probing deep questions related to the complexities involved in human social evolution. In addition to those already discussed, the following are some promising directions for future research.

- *Sex- or age-based division of labor*. What would be the effect of groups developing traditions of varying the level of alloparenting contribution by sex, age, or reproductive status? For example, what if mothers with young were not expected to contribute alloparenting effort?
- *Changes in life history parameters.* In the present model, individuals have the same life history as observed in modern humans, with non-reproductive periods at the beginning and end of life. These non-reproductive periods are unique to humans, however, and may have evolved in the context of cooperative breeding families. By modifying this model we can look at the condition which may have favored changes to the human life history.
- *Parenting strategies.* In this model, it is assumed that the likelihood of an agent being a cooperator is influenced only by the proportion of cooperators in the group. In modifications to the model we can investigate how parenting strategies may introduce other influences.
- *Mate choice strategies.* Current models which consider the evolution of mate choice assume it to be an individual decision, ignoring the evidence that extended families have historically played a large role in mate decisions [73]–[76]. How might different mate choice strategies developed at the group level affect fitness of the group and individual? How do conflicts between individual and family-level preferences play out over the long term?
- *Wealth accumulation, bride prices, and dowries.* Individual or families could accumulate wealth and use it to influence mate choice and the reproductive success of descendants.
- *Institutions of social enforcement.* Individuals within families could punish non-cooperators either directly, by actively taking away resources, or indirectly, by withholding future aid. Institutions of social enforcement likely played an important role in the evolution of human social groups [77], [78].
- *Complex strategies.* Individuals could play probabilistic or contingent strategies to decide whether to cooperate. Families may form alliances, or individuals from different families might form friendship networks to help one another in times of need.

## Supporting Information

Appendix S1 **Detailed model description.**
(DOCX)
