# Supplementary material for: Simulating the Evolution of the Human Family: Cooperative Breeding Increases in Harsh Environments
Source: PLoS One. 2013 Nov 20;8(11):e80753. doi: 10.1371/journal.pone.0080753 (PMC3835414; doi:10.1371/journal.pone.0080753)
Supplement: Appendix S1 — Detailed model description. (DOCX) [file pone.0080753.s001.docx]

**Appendix S1: Model specifics**

**Mathematical functions**

This section will describe all the mathematical functions used in the model.

**1. Genetic quality.** At the initialization of the simulation, each agent *i* is assigned a trait *g_i_* which is drawn from a normal distribution with mean μ and standard deviation σ_0_. The trait of an initialized child *k* with mother *i* is *q_i_* + ε, where the error term is drawn from a normal distribution with mean of zero and a standard deviation of *σ_c_*. Each subsequently birthed child *k* to parents *i* and *j* will have a trait value given by the average of *g_i_* and *g_j_*, plus a normally distributed variation with mean of zero and standard deviation of *σ_c_*. This trait, though normally distributed, will correspond to fitness value that varies between zero and one. Following Boyd and Richerson (1985, ch. 4), let an agent’s genetic quality be given by

 (S.1)

where $\hat{g}$ is the value of the trait with maximum fitness, and *S* controls the intensity of selection. The maximum possible genetic quality was bounded at γ (0 < γ ≤ 1). For the baseline model, γ = 1.

**2. Matchmaking.** Eligible males and females use a probabilistic function to determine whether each will accept his or her date as a partner, which increases with the attractiveness of the potential partner, and becomes less choosy with increasing age. This function has been adapted from previous mate choice models (Kalick & Hamilton, 1986; Smaldino & Schank, 2012) to fit the assumptions of our model. The probability of agent *j* accepting date *i* is given by

$P(j,i)=\left[\left(q_{i}+\varepsilon_{m}\right)^{h}\right]^{(D-d_{j})/D}$ (S.2)

reflecting preferences for mates with high genetic quality. The error term ε*_m_* denotes the error in estimation of preference signals, and is set to zero in the runs presented here. The exponent *h* represent the baseline pickiness in how likely individuals are to choose mates that differ from their ideals, *D* is the number of years between entering adulthood and entering elderhood, and *d_j_* is the number of years elapsed since *j* entered adulthood.

**3. Available resources.** Agents vary in the resources they have at their disposal to contribute to individual and collective parenting, which varies by age and genetic fitness. In our model, children do not contribute to collective parenting. The resources for agent *i* with age *a_i_* is given by

$R_{i}=\left\{\begin{matrix} 0 & \text{if }i\text{ is a child} \\ 2(a_{i}-a_{adult})+r(q_{i}) & \text{if }i\text{ is an adult} \\ 2(a_{elder}-a_{adult})\exp[-k_{r}(a_{i}-a_{elder})]+r(q_{i}) & \text{if }i\text{ is an elder} \end{matrix}\right.$ (S.3)

where *a_adult_* and *a_elder_* are the ages of adulthood (18) and elderhood (50), respectively, *k_r_* = .05 is a decay parameter, and the baseline resources from genetic fitness is *r*(*q_i_*) = 10*q_i_* + 5. Note that while empirical studies have shown there to be sex differences in the ability to collect food resources (Hill & Hurtado, 2009; Pacheco-Cobos et al., 2010), we do not make any assumptions about sex differences here. This is partly for purposes of simplicity, but also because the direct and indirect contributions of males and females to collective parenting may be difficult to quantify.

**4. Childbearing and childrearing costs.** We assume a constant cost of β “resource units” to bear a child. The costs of maintaining each child decreases exponentially as it approaches the age of adulthood. This cost for a child at age *a* is given by

$C=\beta\cdot\exp[-k_{c}a]$ (S.4)

where *k_c_* is a control parameter for the rate of decay, and was set to 0.2.

**5. Death.** Each year an agent has a chance of dying from illness or accidents. This is a function of age and genetics. The probability of death for agent *i* is given by

$M_{i}=\left\{\begin{matrix} \begin{matrix} \delta(q_{i})\cdot\exp\left[\frac{a_{i}}{a_{adult}}\cdot\ln\left(\frac{d(q_{i})}{\delta(q_{i})}\right)\right] \\ d(q_{i}) \end{matrix} & \begin{matrix} \text{if }i\text{ is a child} \\ \text{if }i\text{ is an adult} \end{matrix} \\ \exp\left[\left(\frac{a_{i}-a_{elder}}{a_{elder}}\right)^{\alpha(q_{i})}\cdot\ln\left(2-d(q_{i})\right)\right]-1+d(q_{i}) & \text{if }i\text{ is an elder} \end{matrix}\right.$ (S.5)

The function δ(*q_i_*) = 0.005 – (0.0025)*q_i_* is the baseline child death rate. Once adulthood is reached, the death rate stabilizes between 0.002 and 0.004, depending on genetic quality, using *d*(*q_i_*) = 0.002 – (0.001)*q_i_*, followed by an exponential increase during elderhood. At age 100, the death rate is 100% (i.e., *M* = 1). For elders, genetic quality affects the rate of increase in death rate through the parameter α(*q_i_*)= 3 + *q_i_*.

**State variables and scales.**

In this model, time is represented discretely. Space is not explicitly modeled, other than to have agents belong to families, each of which is on a discrete “patch,” which in turn has no spatial properties beyond a maximum carrying capacity. The state variables in the model are summarized in **Table S1**.

**Process overview and scheduling**

All code was written in Java and scheduled using the MASON simulation environment (Luke et al., 2005). The model proceeds in discrete time steps, and procedures are executed in the following order:

1. MATCHMAKING
   - All unmarried males and females are randomly paired for a “date”. If they mutually accept each other (Eq. S.2), they become mates, and the female leaves her family group and joins that of her husband.
2. FAMILY FISSIONING
   - Families are scheduled in random order. If the family is bigger than *familySplitSize* AND there is an empty patch available, the family splits onto this new patch. Each group will consist of equal numbers of each type of adult, with equal numbers of each sex from each. Children will accompany their mothers.
3. RESOURCE CONTRIBUTIONS
   - Within each family, each adult *i* contributes the proportion of his or her resources, *R_i_*, to the common pot, where the proportion is determined by *i*'s cooperativity.
   - The total resources in the common pot are distributed equally among the married females in the family, and added to any resources each female had left over after her contributions.
4. CHILDBIRTH, CHILDREARING, AND CHILD DEATH
   - - Each mother makes sure she can provide for her extant children. If she cannot, the children will die in order of youngest to oldest until their mother’s resources are sufficient to provide for the remaining offspring.
     - If, after providing for her existing children (if any), there are enough resources to produce a new offspring, she does so. The newborn’s sex and genetic fitness are determined using equations described in section 1.
     - Each child has a nonzero probability of dying, given by Eq. S.5.
     - Each surviving child’s age is increased by one year. If a child reaches the age of adulthood, he or she becomes an independent unmarried adult in his or her mother’s family. The probability that the new adult will be a cooperator is given by the proportion of already existing cooperators in his or her family.
5. ADULT AGING AND DEATH

- Each adult has a nonzero probability of dying, given by Eq. S.5. If the agent does not die, his or her age is increased by one year. If a female with children dies, her children also perish.

**A.4. Design concepts**

- *Emergence*: Family size and composition, proportion of cooperators, and mean genetic fitness emerge from the population dynamics and group-level selection on family groups that survive and produce viable offspring.
- *Evolutionary adaptation*: Agents do not adapt during their lifespan, but individual genetic quality and cooperativity are heritable via genetic and cultural transmission, respectively.
- *Fitness*: Fitness is modeled implicitly: those agents that survive and reproduce most effectively have the highest fitness.
- *Prediction*: Agents lack the ability to predict outcomes of future interactions, though there is an implicit acceptance in the mate choice dynamics that one is aging and therefore mate preferences must be relaxed as an individual approaches elderhood.
- *Sensing*: Agents can assess the genetic quality of potential mates, and families can detect their own group size.
- *Interaction*: It is assumed that potential pairings are assessed from among all families, and that migration to the husband’s family occurs for new brides. Mothers are also assumed to provide care for their offspring, with help from cooperative family contributions. Husbands are not assumed to contribute preferentially to their offspring, but wives are assumed to mate exclusively with their first husbands.
- *Stochasticity*: Pairings for potential marriages are random, and acceptance of a potential mate is probabilistic. Mate choice decisions take many factors into account, therefore we model only an increase or decrease in the probability of a mate choice decision with regard to the genetic factors considered in the model. Death is also stochastic, though related to age and genetic quality. This models the fact that younger and more fit individuals are less likely to die, but time and random events may make skeletons of even the strongest individuals.
- *Collectives*: Children are grouped with their mother, and share her fate. Individuals belong to specific family groups and contribute resources to collective parenting within those groups.
- *Observation*: Simulations were run for 10,000 time steps, and aggregated data was averaged from the final time step across 50 runs for each condition.
- *Initialization*: All runs were initialized according to the default parameters values in **Table S2**. All four age groups were equally represented in both number and sex ratio. Within each category, random ages were assigned. Agents each had a 50% chance of being cooperators, and genetic quality was assigned as described the first section above.

**Table S1.** Overview of state variables.

| **Entity** | **Subfamily** | **State variable** | **Symbol** | **Description** |
| --- | --- | --- | --- | --- |
| Global | Initialization | Number of Families |  | Number of available patches for family groups |
|  |  | Cooperator frequency |  | The proportion of agents initialized as cooperators |
|  |  | Family size |  | Starting number of agents per family group |
|  |  | Family capacity |  | The carrying capacity of each patch |
|  |  | familySplitsSize |  | The family size at which families attempt to fission |
|  |  | Age of adulthood | *a*_adult_ | The age a child becomes an adult |
|  |  | Age of elderhood | *a*_elder_ | The age at which reproduction ends |
|  |  | Cooperator share |  | The fraction of individual resources contributed to the family pot by cooperators |
|  |  | Defector share |  | The fraction of individual resources contributed to the family pot by free-loaders |
|  | Childrearing | Cost of childrearing | β | The cost of producing a child |
|  |  | Child trait SD | σ_c_ | The standard deviation of the error term by which a child’s fitness trait differs from the mean of its parents’ genetic traits |
|  | Genetic Fitness | Genetic mean | μ | The mean value for a normally distributed trait related to fitness |
|  |  | Genetic SD | σ_0_ | The standard deviation for a normally distributed trait related to fitness |
|  |  | G_Optimal | $\hat{g}$ | The value of the genetic trait that maximizes fitness |
|  |  | G_Selection | *S* | The intensity of selection of the genetic  trait |
|  |  | Max genetic quality | γ | The maximum genetic quality |
|  | Match-making | mateError | ε_m_ | Error in estimating mate signal |
|  |  | pickiness | *h* | Baseline pickiness for mate preference |
|  |  | maxDates | *D* | The number of years into adulthood at which max choice becomes arbitrary |
|  |  | Female joins |  | Does female always join male's family group? Random if false. |
|  | Death | Adult death rate |  | The maximum probability of death per year during (non-elder) adulthood |
|  |  | Elder death increase |  | Control parameter for the rate of increase in probability of death during elderhood |
|  |  | Child death rate |  | The maximum probability of death at birth |
| Agent |  | Age | *a_i_* | The agent's age in years |
|  |  | Sex |  | The agent's sex |
|  |  | Family |  | The agent's family group |
|  |  | Genetic fitness | *g_i_* | A variable that influences mortality, resource availability, and sexual attractiveness |
|  |  | Cooperativity |  | How much of his or her individual resources does the agent contribute to collective parenting |
|  |  | Marital status |  | Is the agent married? |
|  | Female only | Children |  | An array of all the female's children |
|  |  | Offspring quality |  | The mean value for the genetic fitness of the agent's offspring, determined by averaging her own trait value with that of her mate |

**Table S2.** Initial and default values for state variables.

| **Subfamily** | **State variable** | **Initial/default value** |
| --- | --- | --- |
| Initialization | Number of Families | 80 |
|  | Cooperator frequency | 0.5 |
|  | Family size | 288 |
|  | Family capacity | 300 |
|  | Family splitsSize | 150 |
|  | Age of adulthood | 18 |
|  | Age of elderhood | 50 |
|  | Cooperator share | 0.9 |
|  | Defector share | 0.1 |
| Childrearing | Child trait SD | 0.3 |
|  | Cost of birth | 100 |
| Genetic Fitness | Genetic mean | 0 |
|  | Genetic SD | 0.5 |
|  | G_Optimal | 1.2 |
|  | G_Selection | 1 |
|  | Max genetic quality | 1 |
| Match-making | mateError | 0 |
|  | pickiness | 3 |
|  | maxDates | 32 |
|  | Female joins | TRUE |
| Death | Adult death rate | 0.002 |
|  | Elder death increase | 3 |
|  | Child death rate | 0.005 |

**References**

Boyd, R., & Richerson, P. J. (1985). *Culture and the evolutionary process.* Chicago: University of Chicago Press.

Hill, K., & Hurtado, A. M. (2009). Cooperative breeding in South American hunter-gatherers. *Proceedings of the Royal Society B*, *276*, 3863-3870.

Kalick, S. M., & Hamilton, T. E. (1986). The matching hypothesis reexamined. *Journal of Personality and Social Psychology, 51*, 673–682.

Pacheco-Cobos, L., Rosetti, M., Cuatianquiz, C., & Hudson, R. (2010). Sex differences in mushroom gathering: Men expend more energy to obtain equivalent benefits. *Evolution and Human Behavior, 31,* 289–297.

Smaldino, P. E., & Schank, J. C. (2012). Human mate choice is a complex system. Complexity, 17, 11–22.

Luke, S., Cioffi-Revilla, C., Sullivan, K., & Balan, G.C. (2005). MASON: A multi-agent simulation environment. *Simulation, 81*, 517–527.
